# Experimental Study on Shear Capacity of Reinforced Concrete Beams with Corroded Longitudinal Reinforcement

**DOI:** 10.3390/ma12050837

**Published:** 2019-03-12

**Authors:** Sun-Jin Han, Hyo-Eun Joo, Seung-Ho Choi, Inwook Heo, Kang Su Kim, Soo-Yeon Seo

**Affiliations:** 1 Department of Architectural Engineering, University of Seoul, 163 Seoulsiripdaero, Dongdaemun-gu, Seoul 02504, Korea; sjhan1219@gmail.com (S.-J.H.); joo8766@uos.ac.kr (H.-E.J.); ssarmilmil@gmail.com (S.-H.C.); inwookheo@gmail.com (I.H.); 2 Department of Architectural Engineering, Korea National University of Transportation, 50 Daehak-ro, Geomdan-ri, Daesowon-myeon, Chungju-si, Chungcheongbuk-do 27469, Korea; syseo@ut.ac.kr

**Keywords:** corrosion, reinforced concrete, shear behavior, bond performance, anchorage

## Abstract

In this study, shear tests were conducted to investigate the effects of longitudinal reinforcement corrosion on the shear capacity of reinforced concrete (RC) members with transverse reinforcement. To this end, a total of eight test specimens were fabricated, and the corrosion rates and anchorage details of rebars were set as test variables. In addition, an accelerated corrosion technique was used to introduce corrosion into the longitudinal reinforcement without corroding shear reinforcement. The test results indicated that the capacities of the specimens in which tension reinforcement was not properly anchored at the ends of the members decreased rapidly at high corrosion rates, whereas the capacities of the specimens in which tension reinforcement was properly anchored by hooks were similar to or higher than those of the non-corroded specimens, despite bond loss caused by corrosion.

## 1. Introduction

In reinforced concrete (RC) structures, reinforcement corrosion is prevented by the strong alkalinity of the concrete cover surrounding the reinforcement [1,2]. However, if carbon dioxide in the atmosphere results in the carbonation of the concrete cover, or if chloride attacks destroy the passive films on the steel reinforcement, corrosion of reinforcement begins, and then the effective sectional area of reinforcement and the bond performance between the reinforcement and concrete drastically decrease [1,2,3]. In this regard, many studies have been conducted on the correlation between the bond performance of reinforcement changed by corrosion and the flexural performance of RC members [4,5,6,7,8,9,10,11,12,13], and several analysis models have been developed so far. Al-Sulaimani et al. [8] and Azad et al. [9] conducted accelerated corrosion tests on tensile reinforcement, and evaluated flexural performance of RC beams with corroded longitudinal tensile reinforcement. Based on the test results, they reported that the flexural strength is reduced by the loss of sectional area of tensile reinforcement at the early stages of corrosion and that the bond failure occurs when the corrosion rate exceeds the critical corrosion rate. Maaddawy et al. [13] carried out an analytical research on flexural behavior of corroded RC members based on bond-slip relationships between corroded reinforcement and concrete, and verified their proposed model by comparing with their test results. In addition, Han et al. [5] suggested a bond failure criterion for the RC members with corroded longitudinal reinforcement.

However, there has been relatively little research on the effects of reinforcement corrosion on the shear capacities of RC members. Higgins and Farrow [14] and Zhao et al. [15] reported that the corrosion in shear reinforcement reduces the shear strength of the RC members. This is caused by a drastic decrease in the sectional area of the stirrups due to pitting corrosion rather than by a decrease in the bond performance between the corroded reinforcement and concrete. EI-Sayed [16] proposed shear strength estimation methods for slender and deep RC beams with corroded stirrups that reflect the loss of sectional area of stirrups and the decrease in effective compressive strength of concrete due to cracks by corrosion.

However, the shear resistance mechanism of the RC members, which is changed by the corrosion of the longitudinal tension reinforcement, has been shown to be more affected by a decrease in bond performance between the reinforcement and concrete than a reduction in the sectional area of reinforcement [17,18,19,20,21]. Azam and Soudki [17,18] reported that the shear capacities of RC members with corroded longitudinal tension reinforcement can be enhanced by about two times the shear capacity of non-corroded RC members due to development of arch action. On the other hand, Jeppsson and Thelandersson [19] mentioned that the shear capacity of corroded RC members decreases as the corrosion rate of longitudinal reinforcement increases, which is caused by the bond loss between corroded reinforcement and surrounding concrete. Xue and Seki [22] reported that the effect of corrosion of longitudinal reinforcement on shear capacity of RC members varies depending on the shear span to depth ratio ($a/d_{s}$). In their test, the shear capacity of corroded RC specimens with $a/d_{s}=2.6$ increased by two times compared with the non-corroded RC specimen, while in the case of corroded RC specimens with $a/d_{s}=4.0$, the shear capacity decreased as the corrosion rate of longitudinal reinforcement increased. Although there have been experimental researches on the shear capacity of RC members with corroded longitudinal reinforcement, their research findings are somewhat conflicted, and, thus, the relationship between corrosion of tensile reinforcement and shear capacity of RC member still requires further investigation. 

Therefore, in this study, shear tests were conducted to examine the shear capacities of RC members with corroded longitudinal reinforcement. For the test, a total of eight RC specimens with transverse reinforcement were fabricated, in which the level of corrosion ($\omega_{corr}=$ 0%, 3%, 8%, and 15%) and anchorage type (straight or hooked) were set as the main variables. To closely examine the effects of corrosion occurring in longitudinal reinforcement on the shear capacities of RC members, the accelerated corrosion technique was used so as to introduce corrosion into the longitudinal reinforcement without any corrosion of the shear reinforcement. Strain gauges were attached to the shear reinforcements of all specimens to measure the strains of transverse reinforcement, and the shear strain distributions of the concrete web were measured using an image-based displacement measurement system [23,24]. In addition, the crack patterns, failure modes, and shear responses of the specimens were analyzed in detail according to the corrosion rate in longitudinal reinforcement.

## 2. Experimental Program

### 2.1. Test Specimens

**Figure 1:**
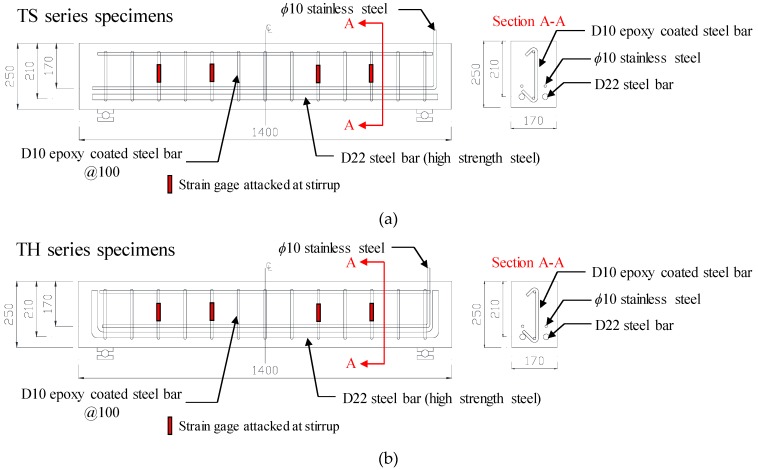
Details of test specimens (Unit: mm). (**a**) TS series specimens; (**b**) TH series specimens.

Table 1 and Figure 1 show the details and material properties of the test specimens, where the test groups are divided into the TS and TH series, respectively. As shown in Figure 1, the longitudinal tension reinforcement of the TS series specimens was anchored with a straight type end, while that of the TH series specimens was anchored with a 90-degree hooked type end. The test specimens of each test group were designed to have four target corrosion rates ($\omega_{corr}=$ 0%, 3%, 8%, and 15%). The widths ($b_{w}$), heights (h), and lengths (L) of all specimens were 170 mm, 250 mm, and 1400 mm, respectively. Two rebars with a diameter of 22 mm were placed on the tension side. To induce shear failure of the member, high strength steel bars with a yield strength of 635 MPa were used for tension reinforcement. In addition, 10 mm diameter shear reinforcements were placed at 100 mm intervals to satisfy the minimum shear reinforcement ratio specified in the ACI 318-14 building code [25], and strain gauges were attached to the shear reinforcement. Since this study aimed to investigate the effects of longitudinal reinforcement corrosion on the shear capacity of RC members with transverse reinforcement, epoxy coated steel bars were used for shear reinforcement to prevent corriosion, and their yield strength was 534 MPa. Meanwhile, for the accelerated corrosion test shown in the following section, 10 mm diameter stainless steel bars were placed inside the test specimens, excluding the reference specimens (TS-0 and TH-0), so that they did not come into contact with the tension reinforcement. The specimens were subjected to steam curing for 24 h after the placement of concrete, then underwent atmospheric curing until the age of 28 days. Finally, the accelerated corrosion test was performed on the specimens. The compressive strength of concrete ($f_{c}{}^{\prime }$) was found to be 56.3 MPa at the time of the shear test.

### 2.2. Accelerated Corrosion Technique

**Figure 2:**
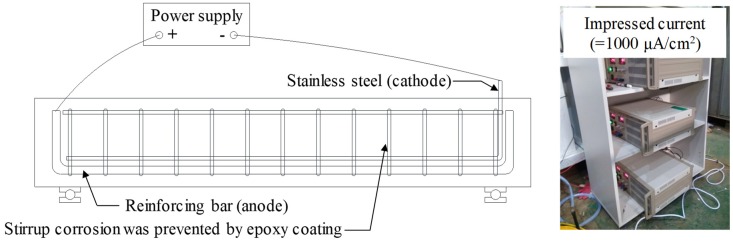
Schematic description of accelerated corrosion test.

Figure 2 shows a schematic description of the accelerated corrosion test. After the age of 28 days, the specimens were precipitated in a 5% NaCl solution, and then dried in air for a week. After the wetting-drying cycle, the specimens were precipitated again in the 5% NaCl solution, and a constant current was provided by connecting the steel bar and stainless steel to direct current (D.C.) power supply with 5.0 A capacity so that the D22 tensile reinforcement could act as the anode and the D10 stainless steel could act as the cathode. Andrade et al. [26] and Al-Harthy et al. [27] reported that the current density ($i_{corr}$) measured in actual RC structures is less than $0.1\;\mu A/{\mathrm{cm}}^{2}$, but previous researchers used current densities ranging from $150\;\mu A/{\mathrm{cm}}^{2}$ to $10,400\;\mu A/{\mathrm{cm}}^{2}$ in the accelerated corrosion test [28]. Lin and Zhao [29] reported that a difference between the corrosion current density in the natural environment and that in the laboratory environment can cause a difference in the type of corrosion products formed on the reinforcement surface as well as the amount of corrosion. They also noted that it is desirable to use as small a $i_{corr}$ value as possible. However, as a lower current density is used, more time is required to achieve the target corrosion rate, thus, making it difficult to derive research results in a limited research project period. Therefore, in this study, $i_{corr}$ was set to $1000\;\mu A/{\mathrm{cm}}^{2}$ based on previous research [28]. The time set to obtain the target corrosion rate ($\omega_{corr}$) was estimated based on Faraday’s law [28], and the times (t) required to obtain the target corrosion rates ($\omega_{corr}$) of 3%, 8%, and 15% were calculated as 5, 14, and 26 days, respectively.

Upon completion of the shear test, the corroded reinforcement was separated from the specimen so as to measure the actual corrosion rate of the longitudinal reinforcement. All of the corrosion products around the tension reinforcement were dissolved using Clark’s solution presented in the ASTM standard G1 [30], and the weight was measured using electronic scales. The actual corrosion rate of the test specimens can be calculated as follows.

(1) $$\omega_{corr}=\frac{m_{0}-m_{1}}{m_{0}}\times 100\%$$

where $m_{0}$ is the initial weight of longitudinal reinforcement, and $m_{1}$ is the weight of longitudinal reinforcement after removing all of the corrosion products.

### 2.3. Shear Test Set-Up

**Figure 3:**
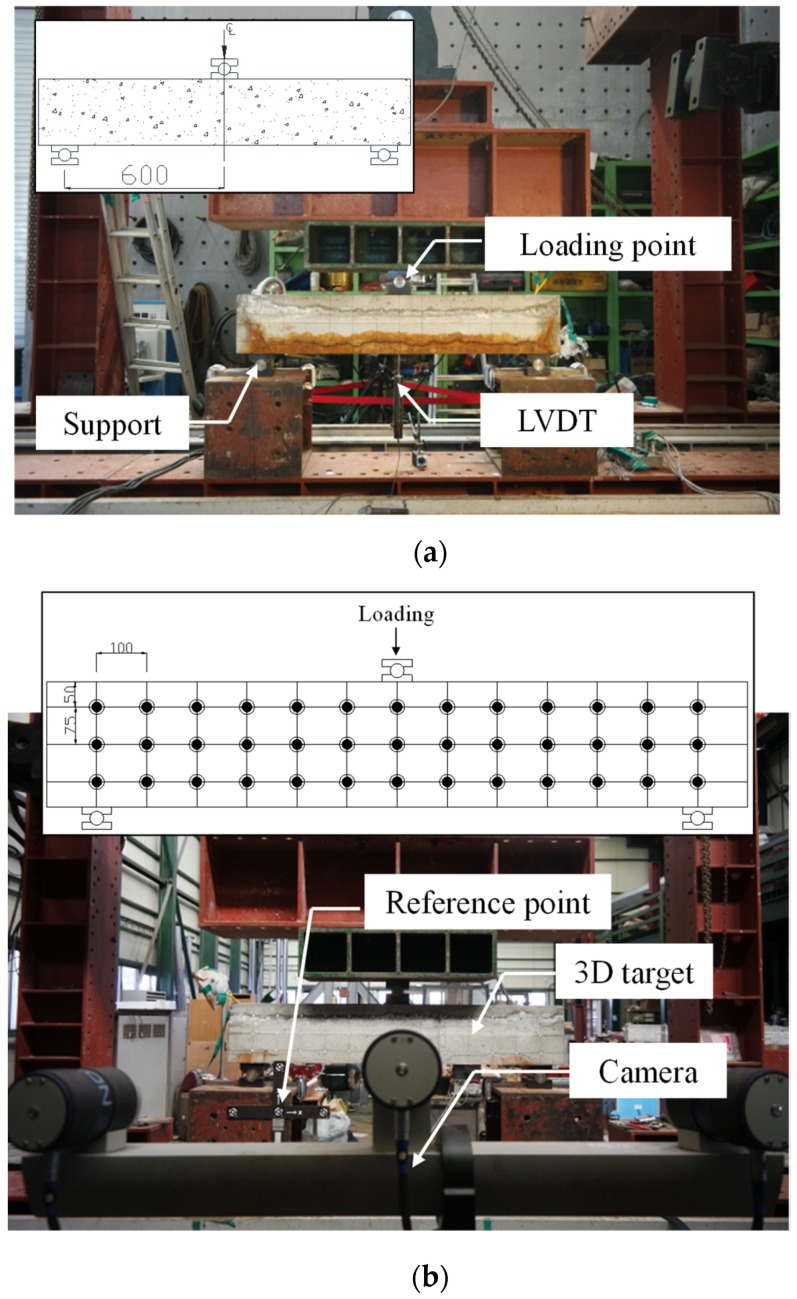
Shear test set-up. (**a**) Test set-up for one-point loading; (**b**) Image-based displacement measurement system.

Figure 3 shows the test setups for the shear test. The shear span (a) was constant for all specimens at 600 mm, and the shear span to depth ratio ($a/d_{s}$) was 2.86. As shown in Figure 3a, the specimens were subjected to one-point loading at the center of the span, and the mid-span deflections of the specimens were measured using a linear variable differential transformer (LVDT) installed on the bottom of the section located at the loading point. In addition, an image-based displacement measurement system [23,24] was used to measure the shear strain distribution of the concrete web, as shown in Figure 3b.

## 3. Experimental Results

### 3.1. Accelerated Corrosion Test Results

**Figure 4:**
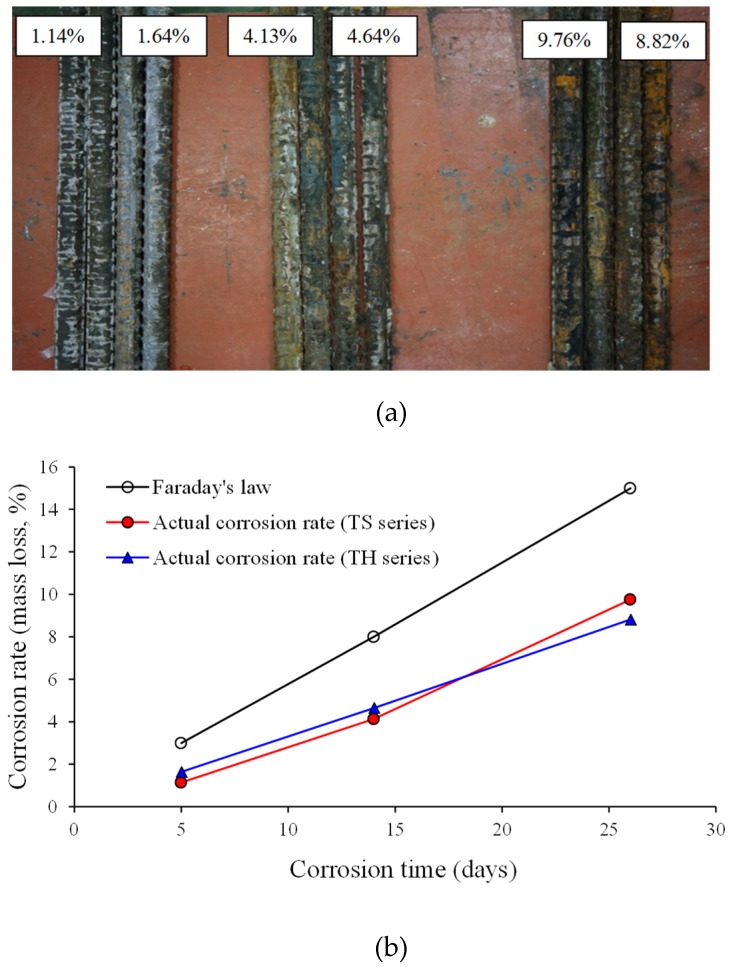
Accelerated corrosion test results. (**a**) Corroded longitudinal reinforcement extracted from test specimens; (**b**) Target and measured corrosion rates of test specimens.

Table 2 and Figure 4 show summaries of the corrosion rates of longitudinal steel reinforcement as calculated using Equation (1). While the target corrosion rates of the specimens were 3%, 8%, and 15%, the measured corrosion rates were 1.14%, 4.13%, and 9.76% in the TS-3, TS-8, and TS-15 specimens, respectively, and 1.64%, 4.64%, and 8.82% in the TH-3, TH-8, and TH-15 specimens, respectively. The reason for why the actual corrosion rates of the specimens are smaller than the target corrosion rates is as follows. As the corrosion of reinforcement progresses, the corrosion product surrounds the reinforcement, thus, interfering with the supply of oxygen (O_2_) and water (H_2_O) needed to form corrosion cells. In addition, as mentioned above, it is estimated that the magnitude of corrosion current density ($i_{corr}=1000\;\mu A/{\mathrm{cm}}^{2}$) used in the accelerated corrosion test is considerably large, which leads to a difference between the corrosion rate calculated from Faraday’s law and the actual corrosion rate. Therefore, to obtain the target corrosion rate, it is desirable to perform the accelerated corrosion test using the current density range ($150\;\mu A/{\mathrm{cm}}^{2}\sim 400\;\mu A/{\mathrm{cm}}^{2}$) as that used by Azam and Soudki [17,18] and Lin and Zhao [29]. 

**Figure 5:**
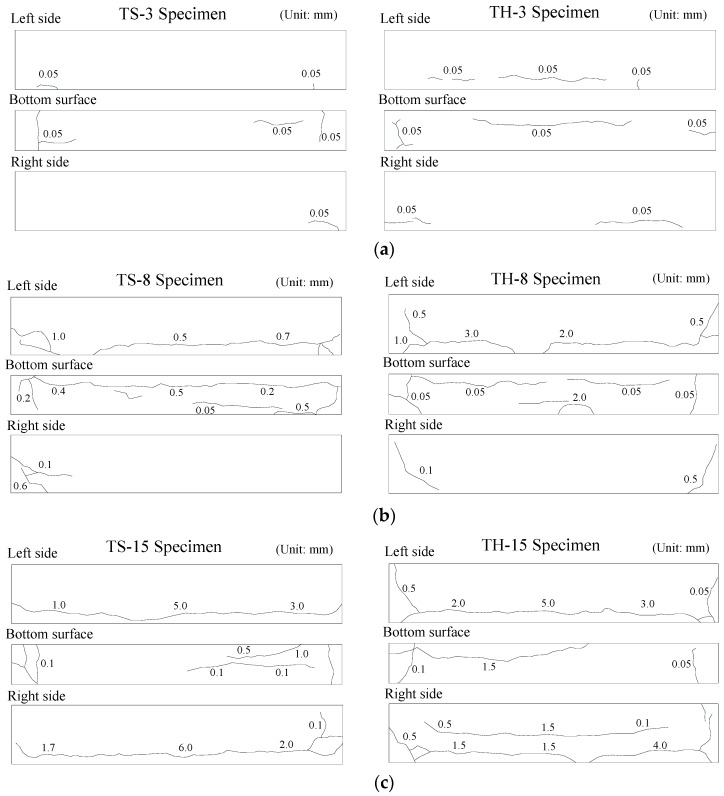
Crack patterns induced by corrosion in longitudinal reinforcement. (**a**) Target corrosion rate of 3%; (**b**) Target corrosion rate of 8%; (**c**) Target corrosion rate of 15%.

Figure 5 shows the crack patterns induced by longitudinal reinforcement corrosion. According to previous studies [8,31], the concrete cover cracks occur in the corrosion rate range of about 1 to 3%, which is the so called corrosion crack. In addition, as the corrosion progresses, the bond performance between reinforcement and concrete decreases sharply with increasing widths of the corrosion cracks. In the TS-3 and TH-3 specimens ($\omega_{corr}$ = 1.14% and 1.64%, respectively) with a target corrosion rate of 3%, only small crack widths of less than 0.05 mm were measured. However, in the TS-8 and TH-8 specimens ($\omega_{corr}$ = 4.13% and 4.64%, respectively), crack widths of more than 0.5 mm were measured along the longitudinal reinforcement layers, and more serious damages to the concrete cover were observed from the TS-15 and TH-15 specimens ($\omega_{corr}$ = 9.76% and 8.82%, respectively), whose crack widths were up to 6.0 mm or more.

### 3.2. Failure Modes of Test Specimens

**Figure 6:**
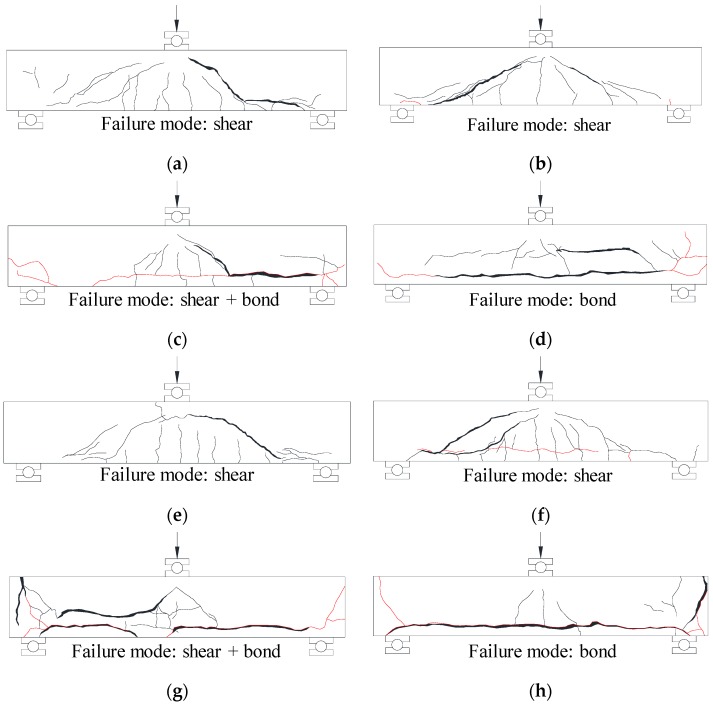
Crack patterns of test specimens after failure. (**a**) TS-0 specimen; (**b**) TS-3 specimen; (**c**) TS-8 specimen; (**d**) TS-15 specimen; (**e**) TH-0 specimen; (**f**) TH-3 specimen; (**g**) TH-8 specimen; (**h**) TH-15 specimen.

Figure 6 shows the crack patterns of the specimens at failure, where the cracks induced by longitudinal reinforcement corrosion (i.e., corrosion cracks) are represented as red lines. The TS-0, TS-3, TH-0, and TH-3 specimens with no corrosion damage or with insignificant damage showed the typical shear failure modes. Meanwhile, the TS-8 specimen ($\omega_{corr}$ = 4.13%) with relatively large corrosion damage exhibited a shear-bond failure mode as shear cracks were connected with corrosion cracks, and the crack width increased rapidly. In the TH-8 specimen ($\omega_{corr}$ = 4.64%), the widths of the corrosion cracks tended to increase with increasing shear crack widths as the load increased, and failure occurred rapidly as anchorage cracks progressed at the end of the member. The TS-15 ($\omega_{corr}$ = 9.76%) and TH-15 ($\omega_{corr}$ = 8.82%) specimens with severe corrosion damage showed the typical bond failure modes. The number of flexural cracks in these specimens was smaller than that in other specimens due to the decreased bond performance between tension reinforcement and concrete. Furthermore, as the load increased, the splitting cracks caused by corrosion became wider, resulting in spalling of concrete cover.

**Figure 7:**
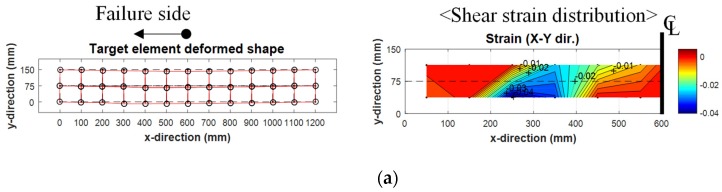

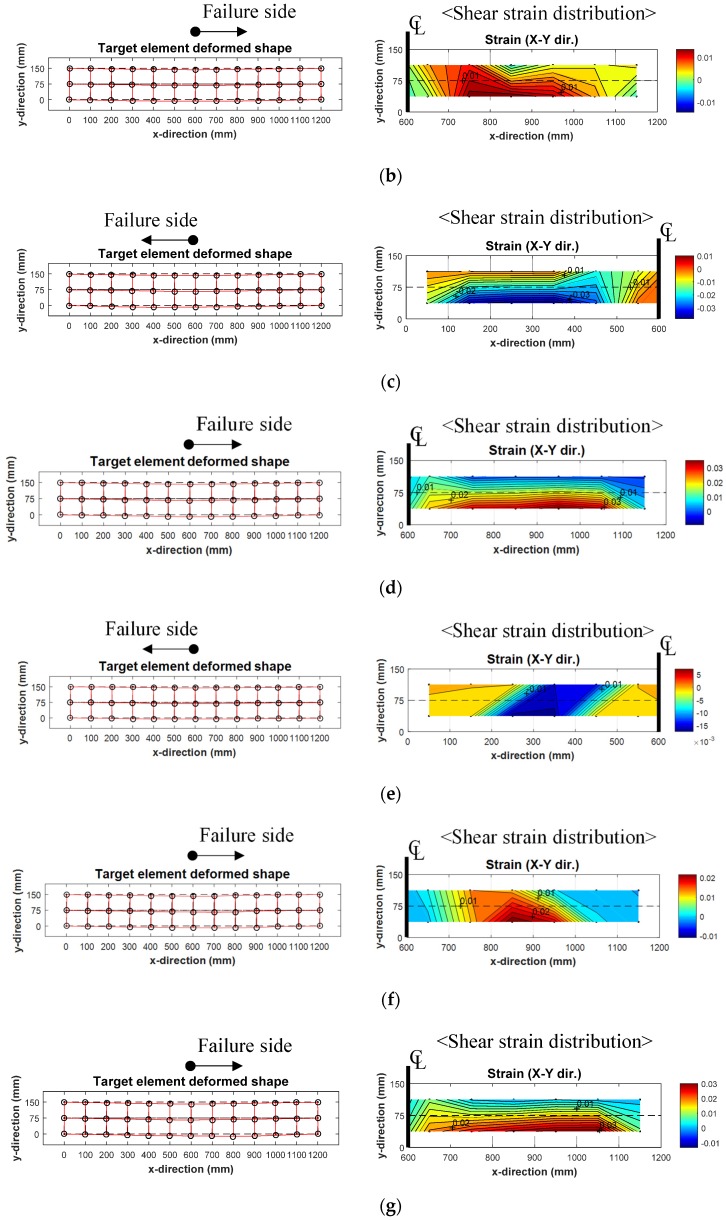

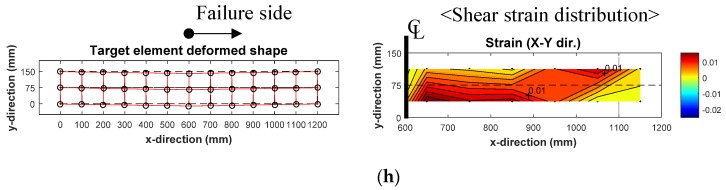
Shear strain distributions at failure loads. (**a**) TS-0 specimen; (**b**) TS-3 specimen; (**c**) TS-8 specimen; (**d**) TS-15 specimen; (**e**) TH-0 specimen; (**f**) TH-3 specimen; (**g**) TH-8 specimen; (**h**) TH-15 specimen.

Figure 7 shows shear strain distributions measured using the relative displacement of the target shown in Figure 3b. For the TS-0, TS-3, TH-0, and TH-3 specimens, in which no tension reinforcement corrosion was introduced or in which corrosion levels were relatively low, the members failed in shear as shear deformation was concentrated in the concrete web. In addition, the TS-8 and TH-8 specimens showed the shear-bond failure modes, resulting from combined deformations due to the shear force and the bond loss. In the cases of the TS-15 and TH-15 specimens with very high corrosion rates, the deformations due to bond loss were found to dominate the failure modes of the specimens.

### 3.3. Shear Behaviors of Test Specimens

**Figure 8:**
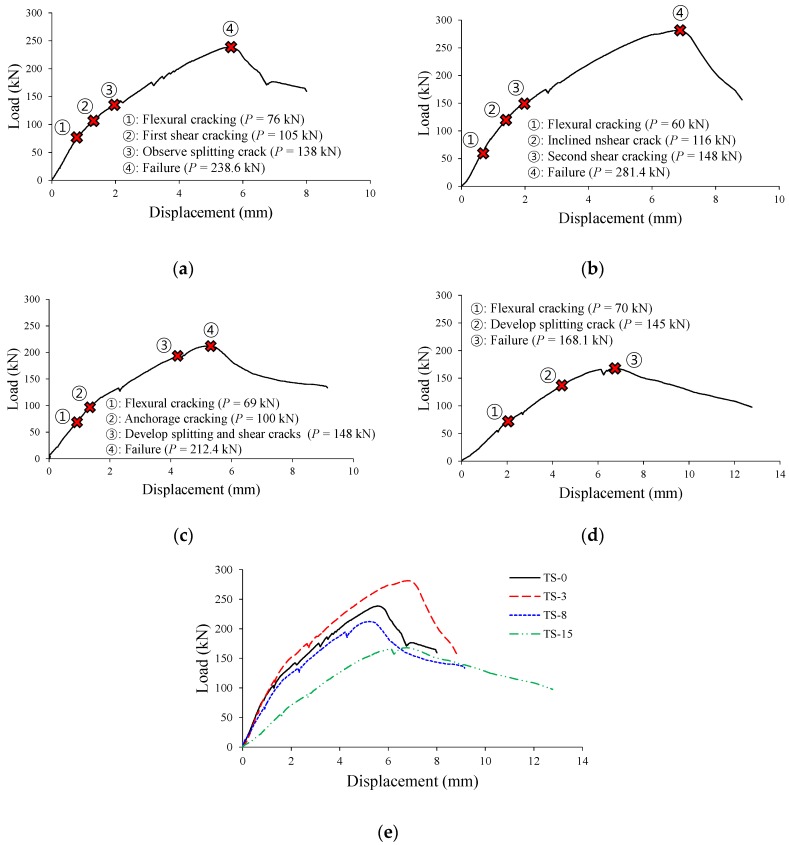
Load-displacement responses of TS series specimens. (**a**) TS-0 specimen; (**b**) TS-3 specimen; (**c**) TS-8 specimen; (**d**) TS-15 specimen; (**e**) Comparison of TS series test results.

**Figure 9:**
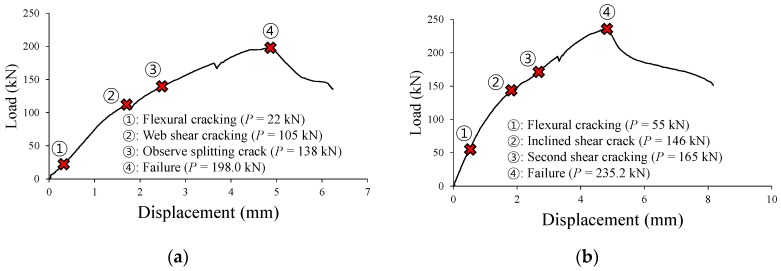

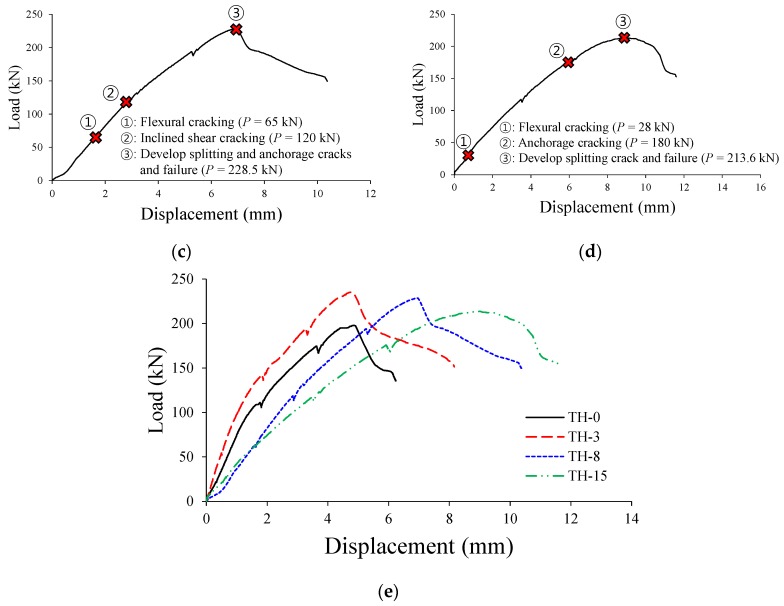
Load-displacement responses of TH series specimens. (**a**) TH-0 specimen; (**b**) TH-3 specimen; (**c**) TH-8 specimen; (**d**) TH-15 specimen; (**e**) Comparison of TH series test results.

Figure 8 and Figure 9 show the shear behaviors of the TS and TH series specimens, while Table 3 summarizes the shear test results. In the TS-0 specimen, the reference specimen of TS series, the initial shear crack occurred at a load of about 105 kN. Then, a horizontal bond crack toward the support was observed at a load of 138 kN, and shear failure occurred at a load of 238.6 kN. In the TS-3 specimen ($\omega_{corr}$ = 1.14%) with a target corrosion rate of 3%, the initial shear crack occurred at a load similar to that of the reference specimen (P = 116 kN), and then underwent shear failure at a load of 281.4 kN. The TS-3 specimen showed a shear capacity of about 18% higher than that of the TS-0 specimen. This is because, at a low corrosion rate of less than 2%, the expansion pressure (i.e., corrosion pressure) of the reinforcement caused by the corrosion contributes to the improvement of bond performance between reinforcement and concrete [8,31], and this phenomenon was observed in the experiment conducted by Lachemi et al. [21]. In the TS-8 specimen ($\omega_{corr}$ = 4.13%) with a target corrosion rate of 8%, a decrease in bond performance due to tension reinforcement corrosion caused a detrimental effect on the member behavior. An anchorage crack was observed at a load of about 100 kN. The splitting crack progressed at the same time when the shear crack occurred at a load of 148 kN, then the member failed at a load of 212.4 kN. The shear capacity of the TS-8 specimen was 11% smaller than that of the TS-0 specimen. The TS-15 specimen ($\omega_{corr}$ = 9.76%) with a target corrosion rate of 15% showed distinct reductions in stiffness and capacity due to corrosion. As shown in Figure 8e, the TS-15 specimen, as compared to the TS-0 specimen, showed very low stiffness from the beginning of the behavior, and the widths of the corrosion cracks became significantly larger with the increasing loads. As a result, bond failure occurred at a load of 161.1 kN, a reduction of 30% as compared to the failure load of the TS-0 specimen. This is because the corroded reinforcement could not exert the tensile stress required to resist the external moment due to bond loss between the reinforcement and concrete.

Figure 9a shows that in the TH-0 specimen, the reference specimen of TH series, a web-shear crack was observed at a load of about 105 kN, a horizontal crack toward the support took place at a load of 138 kN, and then shear failure occurred at a load of 198 kN. As shown in Figure 9e, the TH-3 specimen ($\omega_{corr}$ = 1.64%) showed greater stiffness than the TH-0 specimen from the beginning of the behavior, and also underwent shear failure at a load of 235.2 kN, which is about 19% higher than the load at which the TH-0 specimen underwent shear failure. The reason for why the TH-3 specimen showed greater stiffness and capacity than the TH-0 specimen is that, as mentioned previously, the bond performance between the reinforcement and concrete improves at a low corrosion rate. The initial stiffness of the TH-8 specimen ($\omega_{corr}$ = 4.64%), which has a relatively high corrosion rate, was smaller than that of the TH-0 specimen. The reason for this is that the widths of corrosion cracks in the TH-8 specimen were much larger than those generated in the TS-8 specimen, as shown in Figure 5b. This suggests that the reduction of bond performance in the TH-8 specimen is greater than that in the TS-8 specimen. It is noted that the initial stiffness of the TS-8 specimen was almost the same as that of the TS-0 specimen, as shown in Figure 8e. However, unlike in the TS-8 specimen without a proper anchorage ($\omega_{corr}$ = 4.13%), the failure load of the TH-8 specimen was 228.5 kN, indicating a 15% improvement as compared to the TH-0 specimen with no corrosion damage. This is because the tension reinforcement that has been properly anchored at the end could exert the tensile stress required to resist the external moment, even if the bond performance between the reinforcement and concrete decreased rapidly as the corrosion progressed. The initial behavior of the TH-15 specimen ($\omega_{corr}$ = 8.82%), which has a very high corrosion rate of tension reinforcement, was similar to that of the TH-8 specimen ($\omega_{corr}$ = 4.64%). However, in the TH-15 specimen, inclined shear cracks were not observed during the loading process, as only a few flexural cracks took place at the tops of the corrosion cracks, as shown in Figure 6h. These crack patterns occur when the crack control capability is insufficient, as the bond performance between the reinforcement and concrete is reduced by corrosion. Nevertheless, the capacity of the TH-15 specimen was 213.6 kN, which is about 8% higher than that of the TH-0 specimen. This is because, as mentioned previously, even if the bond performance of the reinforcement decreased due to corrosion, the longitudinal reinforcement could exert tensile stress due to the development of bearing stress at the hooks of the reinforcement anchored at the ends of the member. Therefore, unlike the TS-15 specimen that underwent premature bond failure, the TH-15 specimen exhibited sufficient load-carrying capacity.

**Figure 10:**
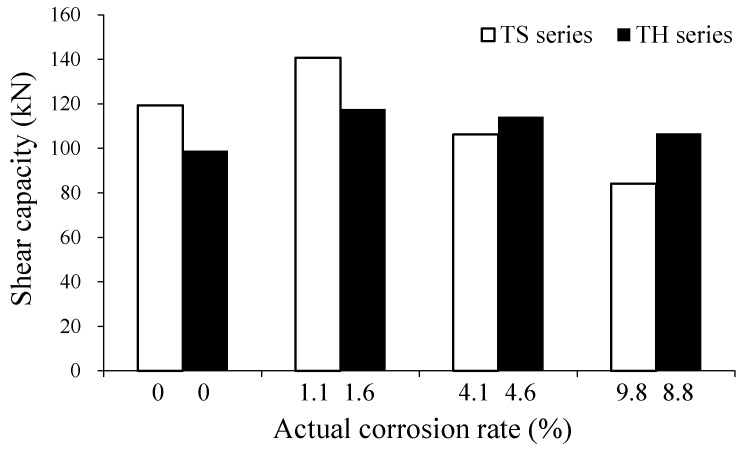
Effects of corrosion rates on shear capacities of test specimens.

As shown in Figure 10, the shear capacities of the TS-3 and TH-3 specimens with low reinforcement corrosion levels (i.e., lower than about 2%) tended to increase by about 20% as compared to those of the reference specimens. However, in the cases of the TS-8 and TS-15 specimens with a high corrosion rate of more than 4%, in which the tension reinforcement had not been properly anchored at the ends of the members, the capacities of the specimens decreased sharply as the corrosion rate of the longitudinal reinforcement increased. By contrast, in the TH-8 and TH-15 specimens in which the tension reinforcement had been properly anchored at the ends of the members in the form of hooks, an increase in corrosion rate did not lead to a capacity reduction of the member, and instead the capacity tended to further increase as compared to the reference specimen.

### 3.4. Measured Strains of Stirrups

**Figure 11:**
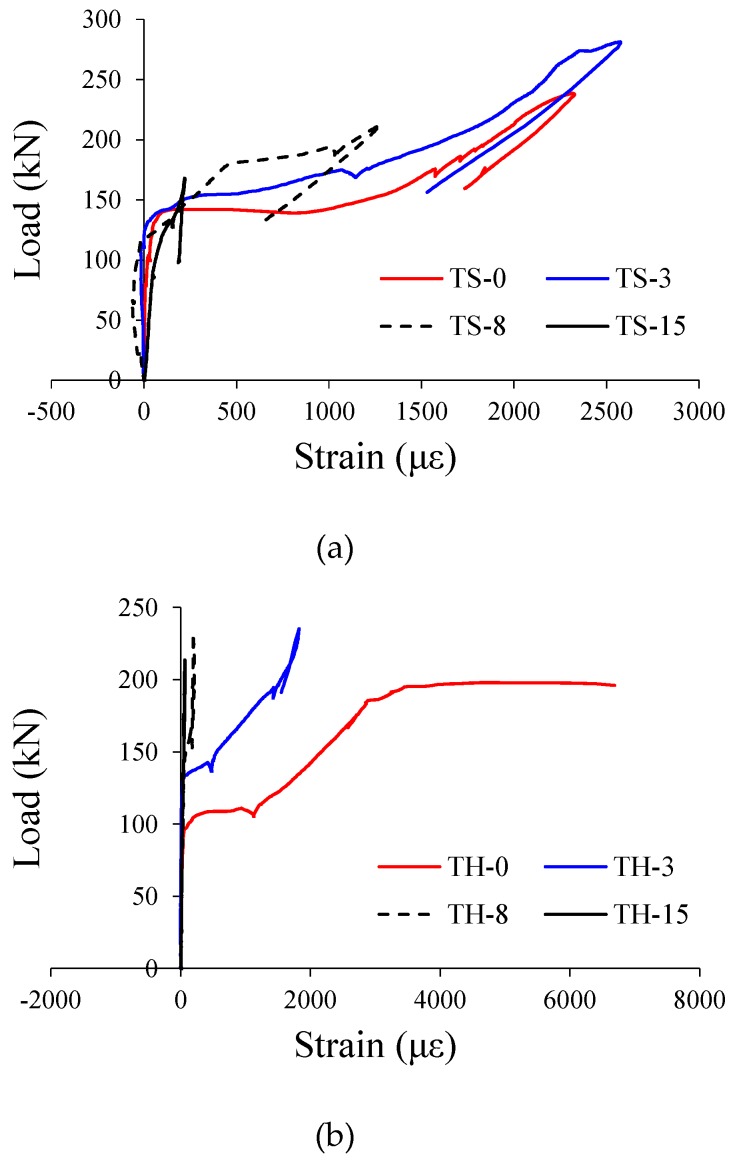
Measured strains of stirrups. (**a**) TS series specimens; (**b**) TH series specimens.

Figure 11 shows the strains measured from the gauges attached to the transverse reinforcement in test specimens. It should be noted that epoxy coated transverse reinforcement was used in this study to prevent corrosion of stirrups. In the TS-0, TS-3, TH-0, and TH-3 specimens, which showed typical shear failure modes, the strains of shear reinforcement increased sufficiently from the shear cracking loads. This suggests that the shear reinforcement contributed to the shear resistance mechanism of the member. By contrast, almost no strains of shear reinforcement or very small strains of shear reinforcement were measured in the TS-15 and TH-15 specimens with very high corrosion rates of tension reinforcement. This is because the failure of these specimens was dominated by the bond loss between the longitudinal reinforcement and concrete rather than the shear.

## 4. Discussion

The TH series specimens exhibited a further increase in the shear capacity of the member as compared to the non-corroded specimen, despite the corrosion of longitudinal reinforcement. Azam and Soudki [17,18] reported that, when tension reinforcing bars were properly anchored at the ends of the member, its shear capacity increased by up to two times as the longitudinal reinforcing bars corroded. They also indicated that this was because the load transfer mechanism of the member was changed from beam action to arch action. Therefore, in this study, the shear capacity of the test specimens was evaluated using a strut-and-tie model (STM) in consideration of the arch-action mechanism and equations for estimating shear capacity presented in the ACI 318-14 building code [25].

The ACI 318-14 code provides the shear capacity equations for slender RC members, as follows:

(2a) $$V_{c}=\left(0.16\sqrt{f_{c}{}^{\prime }}+17\rho_{w}\frac{V_{u}d_{s}}{M_{u}}\right)b_{w}d_{s}$$

(2b) $$V_{s}=\frac{A_{v}f_{vy}d_{s}}{s_{v}}$$

(2c) $$V_{n}=V_{c}+V_{s}$$

where $V_{c}$ and $V_{s}$ are the shear contribution of concrete and stirrups, respectively; $f_{c}{}^{\prime }$ is the compressive strength of concrete (MPa); $V_{u}$ and $M_{u}$ are the external shear force (N) and moment (N·mm) at critical section; $d_{s}$ is the effective depth of reinforcement (mm); $b_{w}$ is the web width (mm); $A_{v}$ is the sectional area of stirrup (mm^2^); $f_{vy}$ is the yield strength of transverse reinforcement (MPa); $s_{v}$ is the spacing between stirrups (mm), and $\rho_{w}$ is the reinforcement ratio, which can be calculated by $A_{s}\left(1-\omega_{corr}\right)/\left(b_{w}d_{s}\right)$ considering the loss of cross-sectional area of longitudinal reinforcement.

In this study, two failure modes (i.e., concrete strut failure or tension tie yield) were considered for the analysis using the STM, as follows:

(3a) $$F_{ns}=0.85\beta_{s}f_{c}{}^{\prime }b_{w}w_{s}$$

(3b) $$F_{nt}=A_{ts}f_{y}$$

(3c) $$V_{n}=\min\left(F_{ns}\sin\theta ,\;F_{nt}\tan\theta \right)$$

where $F_{ns}$ and $F_{nt}$ are the strength of concrete strut and tension ties, respectively;$\beta_{s}$ is the strut effectiveness coefficient, taken to be 1.0; $w_{s}$ is the width of compressive strut (mm), which can be estimated by $l_{b}\sin\theta +w_{t}\cos\theta$; $l_{b}$ is the width of bearing plate (mm); $w_{t}$ is the height of C-C-T nodal zone (mm), and θ is the inclination angle between compressive strut and tension ties (rad). In addition, $A_{ts}$ is the sectional area of corroded tension reinforcement (mm^2^), which can be calculated as $A_{s}\left(1-\omega_{corr}\right)$.

**Figure 12:**
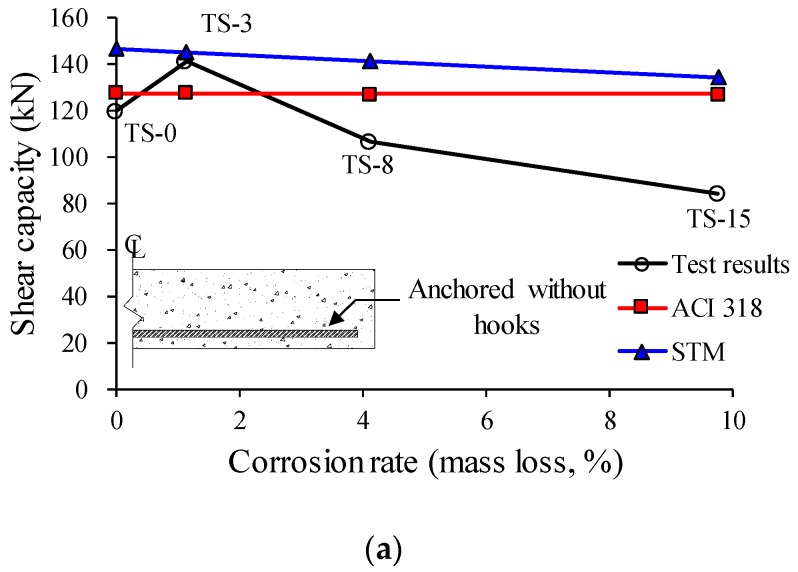

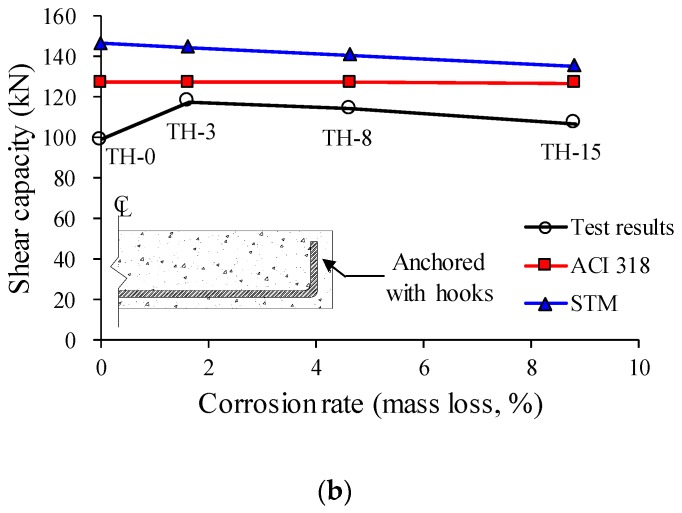
Comparisons of test and analysis results. (**a**) TS series specimens; (**b**) TH series specimens.

Figure 12 shows a comparison of the test and analysis results. The shear capacity equations presented in the current ACI 318-14 building code provided a relatively close evaluation on the shear capacities of RC specimens with a corrosion rate of less than 2%, but significantly overestimated the shear capacities of the TS-8 and TS-15 specimens with a corrosion rate of more than 4%, and the tension reinforcement that had not been properly anchored at the ends of the member. This suggests that the shear capacity is affected more by the reduction of bond performance than by the decrease in the sectional area of tension reinforcement due to corrosion. By contrast, in the TH-8 and TH-15 specimens in which the tension reinforcement had been properly anchored at the ends of the members, there was no substantial difference between the analysis and test results, as the corrosion of longitudinal reinforcement did not significantly affect the capacities of the members due to arch action.

As shown in Figure 12a, the STM provided an excessive overestimation on the shear capacities of the TS-8 and TS-15 specimens. This is because the tension ties of the TS series specimens had not been properly anchored, while a precondition of the application of the STM is that the tension ties should be properly anchored at the ends of the members. Unlike in the cases of the TS series specimens, as shown in Figure 12b, the STM provided a relatively approximate prediction on the tendency of shear capacity changes in the TH-3, TH-8, and TH-15 specimens, in which the tension reinforcement had been properly anchored at the ends of the members. However, even though the arch-action was reflected in the shear transfer mechanism through the application of the STM, the test and analysis results differed because the STM cannot appropriately reflect the changes of bond performance due to the corrosion of tension reinforcement. Therefore, further research is still required to clearly understand the shear capacity of corroded RC members with proper anchorage details, based on which a more proper method for considering the change in the bond performance of the tension reinforcement due to corrosion can be developed.

Meanwhile, there are many retrofitting materials used for strengthening corroded RC members, such as the fabric reinforced cementitious matrix (FRCM) and the textile reinforced mortar (TRM) [32,33]. If the steel wire mesh is applied to the FRCM or TRM, the corrosion would occur with the same mechanism as in the case of reinforcing bars. However, since the bond mechanism between steel mesh and concrete is different from that between reinforcing bar and concrete, the behavior of the structural members with FRCM or TRM whose steel mesh is corroded is expected to be different from the test results reported in this study. Therefore, additional experimental research is required to identify the effects of corrosion on the RC members reinforced with FRCM or TRM.

## 5. Conclusions

In this study, shear tests were carried out to evaluate the effects of corrosion occurring in longitudinal tension reinforcement on the shear capacity of RC members with transverse reinforcement. The shear tests were performed after the introduction of corrosion into the longitudinal reinforcement with the use of an accelerated corrosion technique, and the crack patterns, failure modes, and shear behaviors of the test specimens were measured and analyzed in detail. On this basis, the following conclusions can be drawn:

1. The actual corrosion rate introduced into the tension reinforcement of the specimens differed from the corrosion rate calculated using Faraday’s law. This is because the corrosion products caused by the progress of the reinforcement corrosion interfere with the supplies of oxygen (O_2_) and water (H_2_O) necessary to form the corrosion cell, and the magnitude of the current density ($i_{corr}$) used in the accelerated corrosion test is considerably large. Therefore, to obtain the target corrosion rate in the test, using a small current density ranging from $150\;\mu A/{\mathrm{cm}}^{2}$ to $400\;\mu A/{\mathrm{cm}}^{2}$ is desirable.
2. In the TS series specimens in which the tension reinforcement has not been properly anchored at the ends of the members, the shear capacity of the TS-3 specimen ($\omega_{corr}$ = 1.14%) with a small corrosion rate increased by 18% as compared to that of the TS-0 specimen. This is due to the bond performance of reinforcement that improves at a low corrosion rate of less than 2%. However, the TS-8 ($\omega_{corr}$ = 4.13%) and TS-15 ($\omega_{corr}$ = 9.76%) specimens with high corrosion rates showed 11% and 30% reduced shear capacities, respectively, as compared to the reference specimen. This is attributed to the detrimental effect of the bond loss caused by the corrosion of longitudinal reinforcement on the shear capacities of the members.
3. In the TH series specimens in which the tension reinforcement had been properly anchored at the ends of the members, the shear capacities of the corroded specimens with low corrosion rates as well as those with high corrosion rates were higher than that of the reference specimen. In particular, even the TH-15 specimen ($\omega_{corr}$ = 8.82%), which had the highest corrosion rate, showed an increased capacity of about 8% as compared to the TH-0 specimen. This is because, despite a reduction in the bond performance between corroded reinforcement and concrete, the load transfer mechanism changes from beam action to arch action as the reinforcement has been properly anchored at the ends of the member.
4. The ACI 318-14 code equations overestimated the shear capacities of the corroded specimens and did not provide good predictions over the shear capacity changes of the test specimens in accordance with the corrosion rates. This suggests that the influence of a reduction in bond performance on the shear capacity is more significant than that of a decrease in the sectional area of tension reinforcement due to corrosion.
5. The STM, which reflects the arch-action mechanism, overestimated the shear capacities of the corroded specimens because it failed to reflect the reduction of bond performance between corroded reinforcement and concrete. Therefore, further research is still required to clearly understand the shear capacity of corroded RC members with proper anchorage details, based on which a more proper method to reflect the bond performance of the tension reinforcement due to corrosion can be developed.
6. The research findings of this study indicate that the anchorage details are very important to keep the shear capacity of RC members exposed to deterioration environment.

## Figures and Tables

**Table 1:** Details and material properties of test specimens.

Specimens	*b_w_*	*h*	*d_s_*	*A_s_*	*A_v_*	*s_v_*	*C*	*f_c_′*	*f_y_*	*f_vy_*	*a/d_s_*	*ω_corr_*
	(mm)	(mm)	(mm)	(mm^2^)	(mm^2^)	(mm)	(mm)	(MPa)	(MPa)	(MPa)		(%)
TS-0/TH-0 *	170	250	210	845.5	71.3	100	30	56.3	635	534	2.86	0
TS-3/TH-3 *												3
TS-8/TH-8 *												8
TS-15/TH-15 *												15

* Note: Reinforcing bars in TH series specimens have been properly anchored by hooks. ** Notations: $f_{c}{}^{\prime }=$ compressive strength of concrete (MPa); $b_{w}=$ web width (mm); h= beam height (mm); $d_{s}=$ effective depth of reinforcement (mm); $A_{s}=$ sectional area of non-corroded tension reinforcement (mm^2^); $A_{v}=$ sectional area of stirrup (mm^2^); $f_{y}=$ yield strength of tension reinforcement (MPa); $f_{vy}=$ yield strength of transverse reinforcement (MPa); $s_{v}=$ stirrup spacing (mm); C= cover thickness of concrete; $a/d_{s}=$ shear span to depth ratio.

**Table 2:** Measured corrosion rates of test specimens.

Specimen	Before Corrosion (g)		After Corrosion (g)		Mass Loss (g)		Corrosion Rate (%)		
	Bar 1	Bar 2	Bar 1	Bar 2	Bar 1	Bar 2	Bar 1	Bar 2	Average
TS-3	3803.9	3785.3	3759.7	3742.7	44.2	42.6	1.16	1.13	1.14
TS-8	3809.1	3847.1	3689.1	3650.5	120.0	196.6	3.15	5.11	4.13
TS-15	3856.3	3782.7	3406.9	3485.0	449.4	297.7	11.65	7.87	9.76
TH-3	4619.9	4644.6	4540.4	4572.5	79.5	72.1	1.72	1.55	1.64
TH-8	4631.6	4749.9	4462.9	4481.8	168.7	268.1	3.64	5.64	4.64
TH-15	4750.9	4734.6	4357.6	4291.8	393.3	442.8	8.28	9.35	8.82

**Table 3:** Summary of shear test results.

Specimen	*ω_corr_* (%)	*P_n_* (kN)	*V_n_* (kN)	*∆_mid_* (mm)	Failure Mode	Strength Ratio *
TS-0	0	238.6	119.3	5.54	Shear	1.00
TS-3	1.14	281.4	140.7	6.84	Shear	1.18
TS-8	4.13	212.4	106.2	5.21	Shear + bond	0.89
TS-15	9.76	168.1	84.1	6.76	Bond	0.70
TH-0	0	198.0	99.0	4.82	Shear	1.00
TH-3	1.64	235.2	117.6	4.74	Shear	1.19
TH-8	4.64	228.5	114.3	6.94	Shear + bond	1.15
TH-15	8.82	213.6	106.8	8.99	Bond	1.08

* Shear strength ratio of the specimens to the reference specimens (TS-0 and TH-0 specimens).
